# Connecting cohorts of Finnish biobanks creates a research resource for the study of healthy ageing

**DOI:** 10.1177/14034948241228482

**Published:** 2024-03-04

**Authors:** Niina Eklund, Salla-Maaria Pätsi, Heli Lehtiniemi, Samppa Rohkimainen, Juha Kivelä, Hanna Öhman, Minttu Sauramo, Kyösti Sutinen, Anja Keskinarkaus, Perttu Terho, Tapio Seppänen, Pia Nyberg, Minna Männikkö, Kaisa Silander

**Affiliations:** 1 THL Biobank, Finnish Institute for Health and Welfare, Helsinki, Finland; 2 Arctic Biobank, University of Oulu, Oulu, Finland; 3 Borealis Biobank of Northern Finland, Oulu University Hospital, Oulu, Finland; 4 Center for Machine Vision and Signal Analysis (CMVS), University of Oulu, Oulu, Finland; 5 Auria Biobank, Turku, Finland; 6 Faculty of Medicine, University of Oulu, Oulu, Finland; 7 Research Unit of Translational Medicine, University of Oulu, Oulu, Finland

**Keywords:** Biobank, cohort, electronic health records, research resource, data harmonisation, healthy ageing, genomics

## Abstract

**Aims:**

Connecting cohorts with biobanks is a Finnish biobank collaboration, creating an infrastructure for the study of healthy ageing. We aimed to develop a model for data integration and harmonisation between different biobanks with procedures for joint access.

**Methods:**

The heart of the collaboration is the integrated datasets formed by using data from three biobanks: (a) Arctic Biobank, hosting regional birth cohorts and cohorts of elderly; (b) hospital-affiliated Borealis Biobank of Northern Finland; and (c) THL Biobank, hosting population-based cohorts. The datasets were created by developing a data dictionary, harmonising cohort data and with a joint pseudonymisation process.

**Results:**

The connecting cohorts with biobanks resource at its widest consists altogether of almost 1.4 million individuals from collaborating biobanks. Utilising data from 107,000 cohort participants, we created harmonised datasets that contain attributes describing metabolic risk and frailty for studies of healthy ageing. These data can be complemented with medical data available from Biobank Borealis and with samples taken at hospital settings for approximately 38,000 cohort participants. In addition, the harmonised connecting cohorts with biobanks datasets can be expanded with supplementary data and samples from the collaborating biobanks.

**Conclusions:**

**The connecting cohorts with biobanks datasets provide a unique resource for research on ageing-related personalised healthcare and for real-world evidence studies. Following the FAIR principles on findability, accessibility, interoperability, and reusability, the reused and harmonised datasets are findable and made accessible for researchers. The same approach can be further utilised to develop additional datasets for other research topics.**

## Introduction

We are living in an era in which the proportion of aged people among the global population is growing faster than ever in history due to prolonged life expectancy. According to the World Health Organization (WHO), in 2020 the global number of people over 60 years has surpassed the number of children under 5 years [1]. Although global population and life expectancy are still rising [2], the total population of the European Union, for example, is expected to decline due to low birth rate, presenting a shift in the population structure [3]. More research is needed to explore whether longer life expectancy comes with better functionality [4] or a life with poor health.

According to WHO, healthy ageing is defined as ‘the process of developing and maintaining the functional ability that enables wellbeing in older age’ [5]. Furthermore, ageing can be described as a progressive state of degeneration with underlying molecular changes that lead to chronic diseases [6]. One phenotype of ageing is a clinical condition called frailty. It is characterised as a vulnerability leading to impaired adaptations to external stressors and resulting in adverse outcomes. The increased number of elderly people requires preventive and supportive measures to enable healthy ageing [3, 7].

Finland has a long tradition of monitoring and promoting the population’s health and wellbeing through studies of birth cohorts and population-based health examination surveys, such as the Northern Finland Birth Cohort (NFBC) by the University of Oulu, and health examination surveys conducted by the Finnish Institute for Health and Welfare (in Finnish: Terveyden ja hyvinvoinnin laitos; THL) [8–12]. These cohorts contain extensive data on lifestyle and health status collected through questionnaires and interviews, clinical data measured during clinical visits, and biomarker data derived from collected samples. The health examination surveys and birth cohorts, as well as clinical samples and data from hospital biobanks are available for research through Finnish biobanks, under the Finnish Biobank Act [13, 14].

## Aims

The connecting cohorts with biobanks (CoCoBi) project was a 4-year (2017–2020) Academy of Finland infrastructure project that aimed to create a resource for the study of healthy ageing by harmonising health and lifestyle data from several Finnish birth and population cohorts, and exploring the availability of clinical data from a hospital biobank to enrich the cohort data. These kinds of data are stored by different institutes, in a variety of silos employing various electronic databases, and in formats which do not facilitate data integration from multiple sources. Here we present our work combining and providing a multifaceted study resource for enabling more targeted research of healthy ageing. Through the joint infrastructure established in this project we aimed to create models for harmonisation and integration of data between different biobanks, along with procedures for joint access.

## Materials and methods

### Cohorts included in the CoCoBi project

The cohorts included in the CoCoBi project are presented in Table I.

**Table I.** Description of the biobank cohorts included in the CoCoBi project.

Biobank	Cohort	Inclusion criteria	*N*	Sample/data collection years
Biobank Borealis	FMC	Pregnancy	Approximately 2 million pregnancies of approximately 990,000 women	1983–2016
Biobank Borealis	Prospective hospital collections	Hospital patients	Ongoing collection (approximately 42,000 consents by the end of 2021)	Continuous sample and data collection. The availability year of electronic health data varies depending on the data type
Biobank Borealis	Pathology collection	Diagnostic formalin-fixed paraffin embedded tissue samples	1.8 Million samples from 0.5 million individuals	OUH 1979–2013; central hospitals early 1990s–2013
THL Biobank	FINRISK 1992–2012	Representative, cross-sectional population-based samplings of working-age population in 4–6 large geographical areas in Finland	34,570	1992, 1997, 2002, 2007, 2012
THL Biobank	Health 2000/2011	Population representative sampling of persons aged 30 years and over living in mainland Finland. Follow-up on persons aged 29 years and over	8600	2000–2001, 2011–2012
Arctic Biobank	NFBC 1966	Mothers with expected date of delivery 1.1.1966 to 31.12.1966	At baseline 12,055 mothers and 12,231 children	1966, 1967–1968, 1980, 1997–1998, 2012–2014
Arctic Biobank	NFBC 1986	Mothers with expected date of delivery 1.1.1985 to 30.6.1986	At baseline 9362 mothers and 9479 children	1985–1986, 1986–1992, 1993–1994, 2001–2002, 2019–2020
Arctic Biobank	Oulu 1935	Individuals born in 1935 and living in Oulu, Finland in 1990	At baseline 831	1990–1992, 1996–1997, 2007–2008, 2013–2015
Arctic Biobank	Oulu 1945	Individuals born in 1945 and living in Oulu, Finland in 2000	At baseline 993	2001–2003, 2012–2013

FMC: Finnish Maternity Cohort; NFBC: Northern Finland Birth Cohort; OUH: Oulu University Hospital.

Biobank Borealis is one of six hospital-affiliated clinical biobanks in Finland, covering the Northern part of Finland [15]. Borealis’ sample collections include: (a) diagnostic sample archives transferred from the pathology departments of the Oulu Uni-versity Hospital (OUH) and four central hospitals; (b) collections of prospective samples (serum, plasma, DNA, formalin-fixed paraffin embedded (FFPE) and fresh frozen tissue samples, and cerebrospinal fluid); as well as (c) the Finnish Maternity Cohort (FMC), a nationwide collection of serum samples taken from pregnant women for routine screening of congenital infections during the latter part of the first trimester or the early weeks of the second trimester [16].

The THL Biobank cohorts included in the CoCoBi project were the National FINRISK Study, a series of cross-sectional population surveys carried out every 5 years from 1992 to 2012 [10], and the Health 2000 and 2011 Surveys, a comprehensive health examination survey conducted in 2000 and a re-examination in 2011 [11, 12]. Both cohorts include rich information on lifestyle and health status collected at baseline through interviews and questionnaires, clinical examination data, biomarker data obtained from samples collected during the baseline visit, genomics, and other omics data.

Four birth cohorts from the University of Oulu were included in the project. Two longitudinal and prospective birth cohorts of women and newborns, NFBC1966 and NFBC1986, collected at 20-year intervals from the two northernmost provinces of Finland, Oulu and Lapland [8, 9, 17]. Data from cohort participants have been collected on a regular basis since the antenatal period by health care records, questionnaires, and clinical examinations. This allows study of the life-course determinants and pathways to health and diseases, exploring the role of social, genetic, and environmental factors. Data have also been collected on participants’ parents and offspring. In addition, two longitudinal birth cohorts of the elderly, OULU1935 and OULU1945 [18], with data on ageing and health were included in the project. These cohorts are administered by the University of Oulu, where Arctic Biobank was established in 2020 to host them. For terminological clarity Arctic Biobank will be used here onwards when referring to the cohorts at the University of Oulu.

### Data sharing between the biobanks

**Figure 1.**
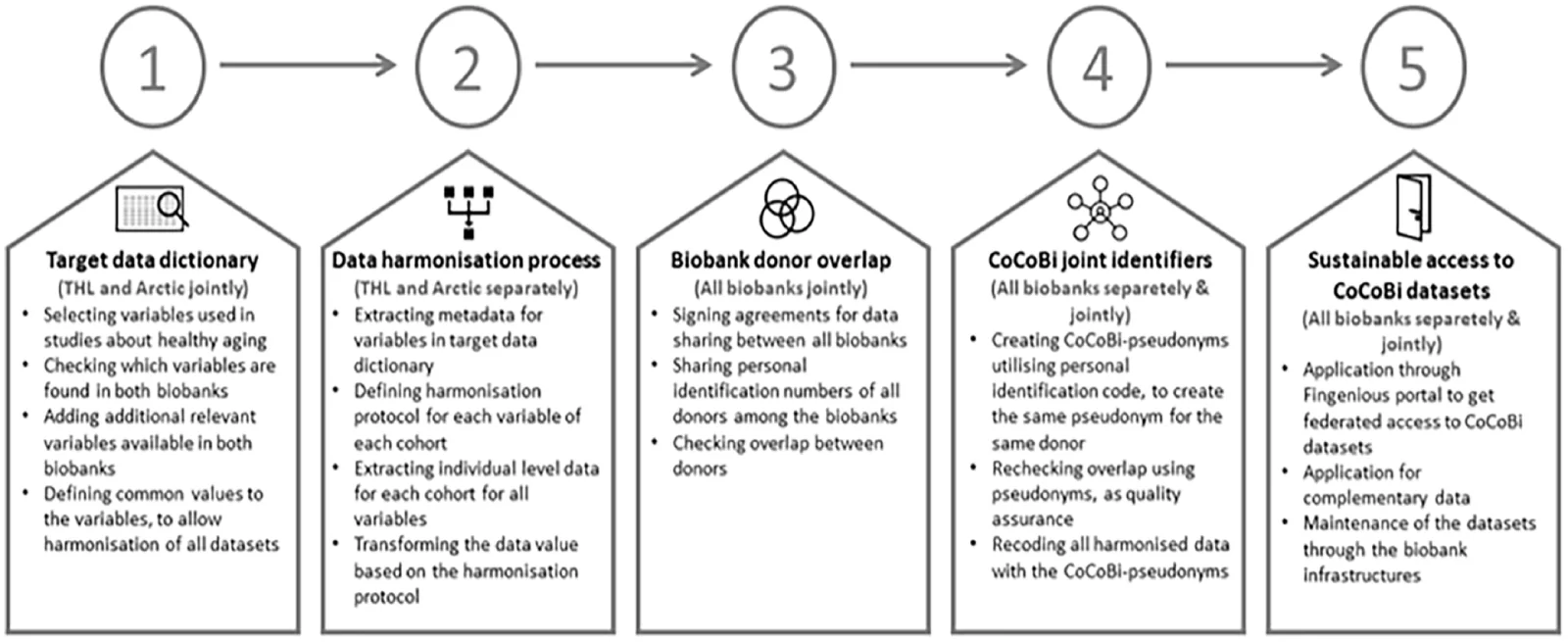
Description of the processes resulting in jointly coded and harmonised datasets in the connecting cohorts with biobanks (CoCoBi) project.

Data sharing was considered for two purposes: (a) analysing the extent to which the participating biobanks have samples and data from the same sample donors; and (b) building up harmonised and jointly coded datasets of all sample donors to be provided to researchers with the approved biobank project. To identify sample donors from whom there are samples and data in more than one biobank, we shared personal identification code (PIC) information of each sample donor in the sample collections included in this project. To enable sharing of PICs, agreements were made separately between all participating organisations, a total of three data sharing agreements including appendices on details and terms of processing of personal data (Figure 1).

### Pseudonymisation to enable data integration

To identify samples and data from the same individuals and to form a joint integrated data resource, the unique Finnish PICs of sample donors from all cohorts were compared in THL and Borealis biobanks (Figure 1). Once overlapping sample donors were identified, PICs were pseudonymised in a joint effort between the three collaborating biobanks. The technical solution for performing joint pseudonymisation was provided by Auria Biobank with a pseudonymisation tool using the MD5 message digest algorithm with salt included. MD5 is a commonly used hash function, which can be used to verify data integrity via checksum functionalities [19]. The Auria Biobank pseudonymisation tool is based on a similar solution developed jointly by Finnish biobanks in the Isaacus Digital Health HUB unpublished preproduction project for breast cancer research. By this method, we could ensure that samples and data from individuals found in two or more cohorts were pseudonymised with the same unique ID.

Each biobank conducted the pseudonymisation of the PICs by separately accessing the same pseudonymisation tool. The PIC lists were uploaded into the pseudonymisation application along with the hash key, and ID key files with PICs and corresponding pseudonyms were created. The uploaded ID lists were automatically deleted from the application working memory after the conversion was completed to ensure biobank sample donor data security and privacy. After all biobanks had completed the pseudonymisation process, a data integrity check for the pseudonymised identifiers was performed by reassessing the overlap between sample donors in the different biobanks using the pseudonyms.

### Data dictionary and data harmonisation protocol

To create a data resource for the study of healthy ageing, we utilised two existing University of Oulu research projects that focused on healthy ageing, metabolic risk factors and frailty, named Frailty and DynaHealth [18, 20]. Selected relevant data categories from DynaHealth consisted of psychosocial factors, glycaemic health data, type 2 diabetes, and cardiovascular disease risk factors. Non-communicable diseases from DynaHealth were stroke, coronary heart diseases, dementia, and Alzheimer’s disease. Three different frailty measurements, frailty phenotype, frail scale and frailty index were included from the frailty study, and were of the following categories: sociodemographic factors, exercise, nutrition, weight loss, anthropometric information and blood pressure, quality of life assessment, and diseases associated with old age. We formed a data dictionary of the target variables and their values, which was then used as the base for the joint datasets created and harmonised in this project (Figure 1). We merged similar variables used in both studies to form a data dictionary for the CoCoBi project, which is made available online [21]. Furth-ermore, we included metabolomic biomarkers available for THL and NFBC cohort participants. To make the data dictionary more informative to a broader user base, we added descriptive metadata to the target variables in the data dictionary. The additional metadata include enumerations for data classes, measurement units of quantifiable variables, measurement protocol descriptions, and basic taxonomy to group similar variables. Also, variable labels were adjusted to be more informative and uniform. The whole dictionary was translated into English.

Once the data dictionary was formed, we proceeded to data harmonisation. The variables suitable for harmonisation for each sample collection belonging to a specific cohort were extracted from Arctic and THL biobanks' databases. There was a total of 11 different sample collections among the CoCoBi cohorts. The data were harmonised locally, each biobank harmonising data for their cohorts using SAS (version 9.4) and SPSS (version 21.0) statistical software, and creating a specific harmonisation protocol for each variable of each sample collection. We evaluated the harmonisation process throughout the project, and when necessary we updated it and the data dictionary to form as uniform data as possible.

### Enrichment of the CoCoBi datasets by hospital and register data

Biobank Borealis has access to a broad spectrum of electronic health record (EHR) data for all biobank-consented individuals, who have an outpatient/inpatient visit to OUH. In this study we focused on the following data categories: demographic data, the International Classification of Diseases, 10th revision (ICD-10) diagnoses [22], particularly diagnoses related to frailty and ageing [23], and the morphology and topology SNOMED codes (SNOMED; the Systematic Nomenclature of Medicine [24] coded diagnoses and organs, structured tables, and free text data) of the pathology diagnostic tissue archives. The amount and nature of clinical information varies, depending on the specific subcollection within Biobank Borealis.

In addition to the data existing in the biobanks’ databases, health-related information about all biobank sample donors in the CoCoBi datasets can be obtained from national social and health registers, which are continuously updated. The Finnish Social and Health Data Permit Authority Findata issues permit access of this register data [25].

## Results and discussion

### Access to the CoCoBi datasets

In this project, we have created comprehensive harmonised datasets from three different Finnish biobanks that can be jointly used for the study of healthy ageing. This was made possible through recoding the harmonised datasets with pseudonyms, which are uniquely made from PICs, producing the same pseudonym for the given PIC. A federated approach in providing researchers access to the harmonised datasets was taken to ensure the proper management and protection of the privacy of the sample donors, and to comply with the European general data protection regulation [26], and with the Finnish Biobank Act [14], which requires that each biobank should have control of the samples and sample-related data which it hosts. Federated data access means that each participating organisation controls access to its own data, and material transfer agreements for a given research project must be made with all owner organisations.

Throughout the data integration process FAIR principles on findability, accessibility, interoperability, and reusability were applied [27]. The existing research data within CoCoBi follow these principles and can be accessed via single application through the Fingenious® Service [28] provided by the Finnish Biobank Cooperative FINBB(Figure 1).

### Overlap and demographics of the CoCoBi datasets

**Figure 2.**
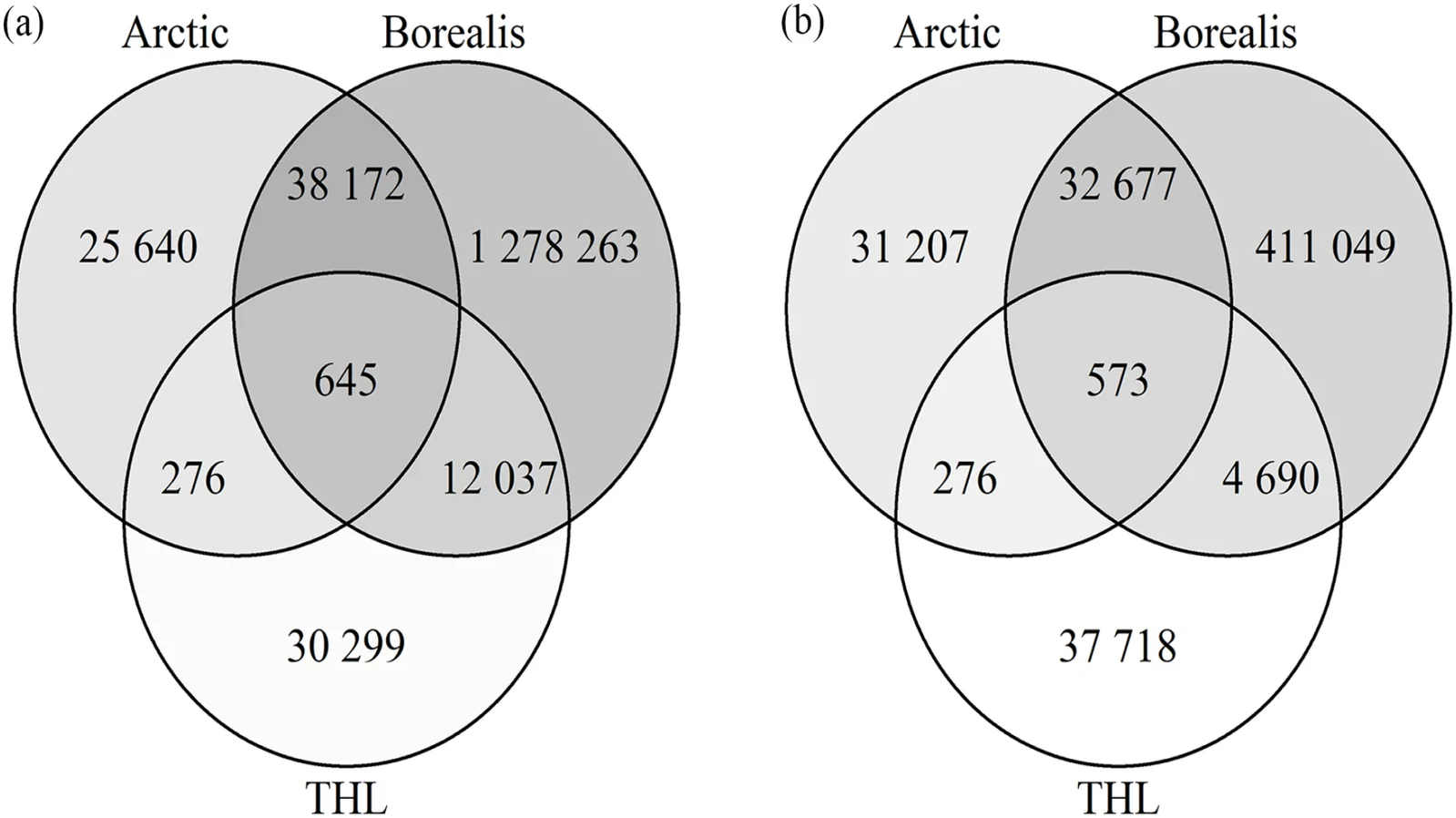
Overlapping subjects in the CoCoBi datasets. (a) All sample donors in Borealis, THL and Arctic Biobanks included. (b) the Finnish Maternity Cohort (FMC) sample collection excluded.

At its widest the CoCoBi dataset has 1.39 million unique sample donors from three biobanks. Observed overlaps among the sample donors are depicted in a Venn diagram (Figure 2). In the core of the joint datasets are 645 individuals whose samples were present in all three biobanks. The overlap of sample donors between all three biobanks is shown in Figure 2(a). The high overlap between Arctic and Borealis biobanks is due to the same geographical location in northern Finland, while the cohorts in the THL Biobank are nationwide health surveys. When the large nationwide FMC collection in Biobank Borealis is not included (Figure 2(b)), a significant decrease is seen in the overlap between Biobank Borealis and THL Biobank.

**Figure 3.**
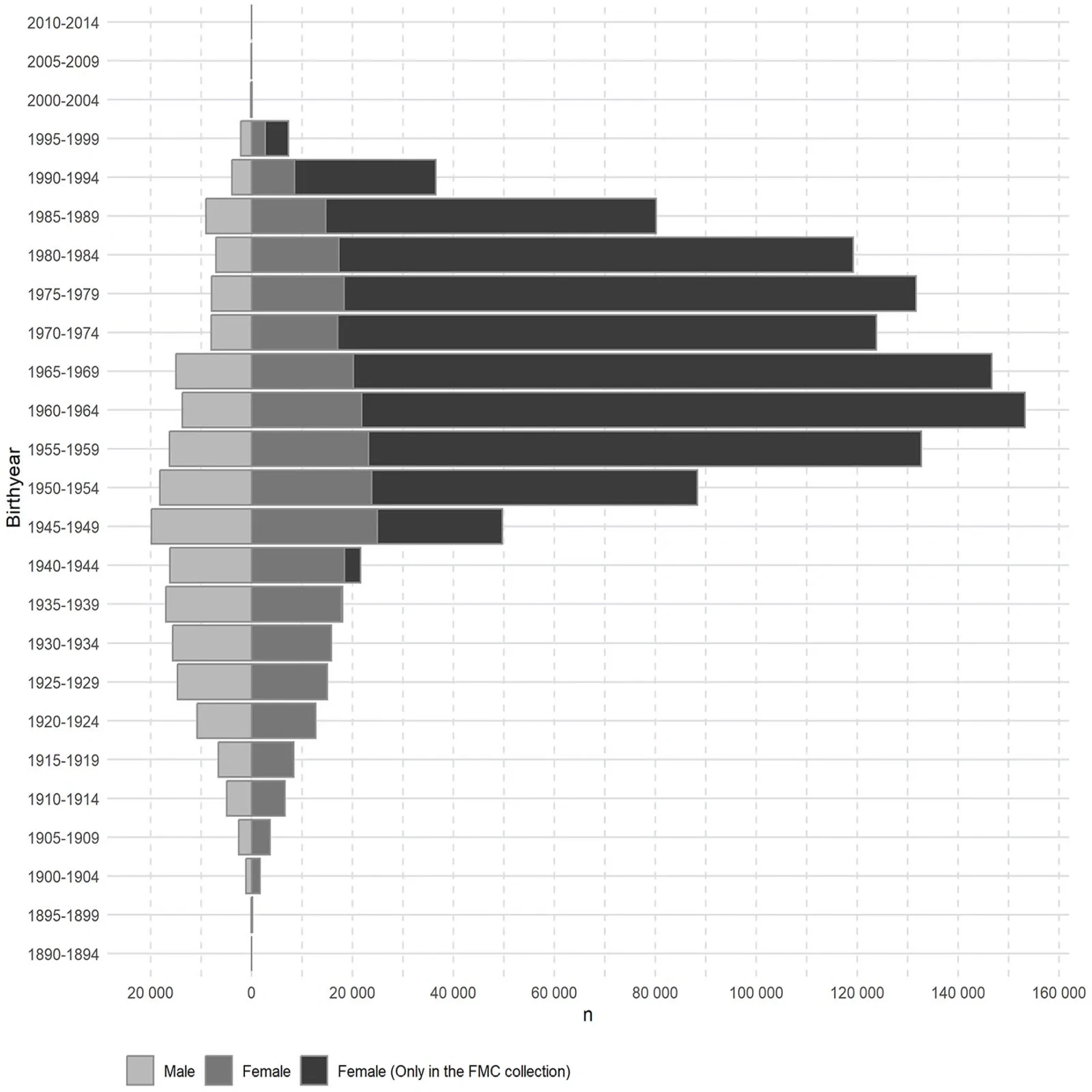
Demographics of the connecting cohorts with biobanks (CoCoBi) dataset. The effect of the Finnish Maternity Cohort (FMC) in the whole dataset is shown by indicating those women with a sample only in the FMC collection (female (only in the FMC collection)).

The overall demographics of the CoCoBi datasets show that more than 518,000 individuals in the dataset were over 60 years old and more than 262,000 were over 70 years at the time of dataset completion in 2020 (Figure 3). Women are overrepresented due to the FMC, which can be seen especially in the younger age groups. Altogether, there were over 38,000 individuals with samples or data in more than one biobank when excluding FMC and over 51,000 when including the FMC.

### Harmonised cohort datasets

The CoCoBi datasets harmonised in this study between THL and Arctic biobanks’ cohorts include 361 variables selected to support the study of healthy ageing [21]. The dictionary includes 120 variables on healthy ageing and 241 nuclear magnetic resonance (NMR) metabolomic data variables. Approximately 33–57% of healthy ageing variables were found from FINRISK 1992–2012 and Health 2000/2011 cohorts, and 22–91% variables were found from NFBC and OULU cohorts. NMR data are available for all cohorts except FINRISK 1992. The availability of variables for harmonisation varied from collection to collection as some data categories had not included all collection time points or for all cohorts – that is, cognitive tests and specifically aging-related diseases were included only in the cohorts targeting older people. For any given cohort, the data that were successfully harmonised are almost complete with only a few missing values.

The easiest variable groups to harmonise between cohorts were basic information on the research participants (i.e. age, sex, marital status), measurements (i.e. physical measures, blood pressure, laboratory and NMR measurements) and existing illnesses. More complicated variables to harmonise were certain sociodemographic factors (i.e. education, occupation) and questions based on strict protocols, such as the RAND 36-item Short Form Survey about work capacity and psychological factors [29], and 15D, the health-related quality of life instrument variables about general health [30]. The complexity in harmonising variables based on these standard questionnaires originates from data collections: even though the variables are based on certain standards, the attributes can be adjusted to data collectors’ own needs, can be only loosely related to the standard, or can contain influences from multiple standards. Therefore, we also adjusted the data dictionary accordingly to enable data harmonisation.

### Complementary data from cohorts and sample availability

For the current project, we harmonised variables that are related to healthy ageing and metabolic health. However, samples and various other data, such as genomic data, are available for cohort participants through THL Biobank and Arctic Biobank. These additional data categories, as well as Biobank Borealis’ FMC collection data, are described in Supplemental Table 1.

Each of the cohorts included in the project also has its own unique datasets. For the NFBC participants, data are available from the gestational period and are complemented by rich longitudinal data collected from birth onwards at various time points during their life course, and in several specific substudies focusing, for example, on oral health [16]. Longitudinal samples and data are available for 4200 participants of the Health 2000 and 2011 Surveys, collected 11 years apart, and for 1300 participants of the FINRISK 2007 study, re-examined in 2014.

FMC offers the possibility to do research projects requiring serial serum samples from consecutive pregnancies, the number of samples from at least three pregnancies being over 260,000. Data are also available from mother–daughter pairs and a linkage of mothers to the NFBC1986 cohort is possible. The data of the FMC maternal collection in Biobank Borealis include basic pregnancy information and various biomarker measurements from previous studies.

### Complementary data from hospital EHRs and sample availability

**Figure 4.**
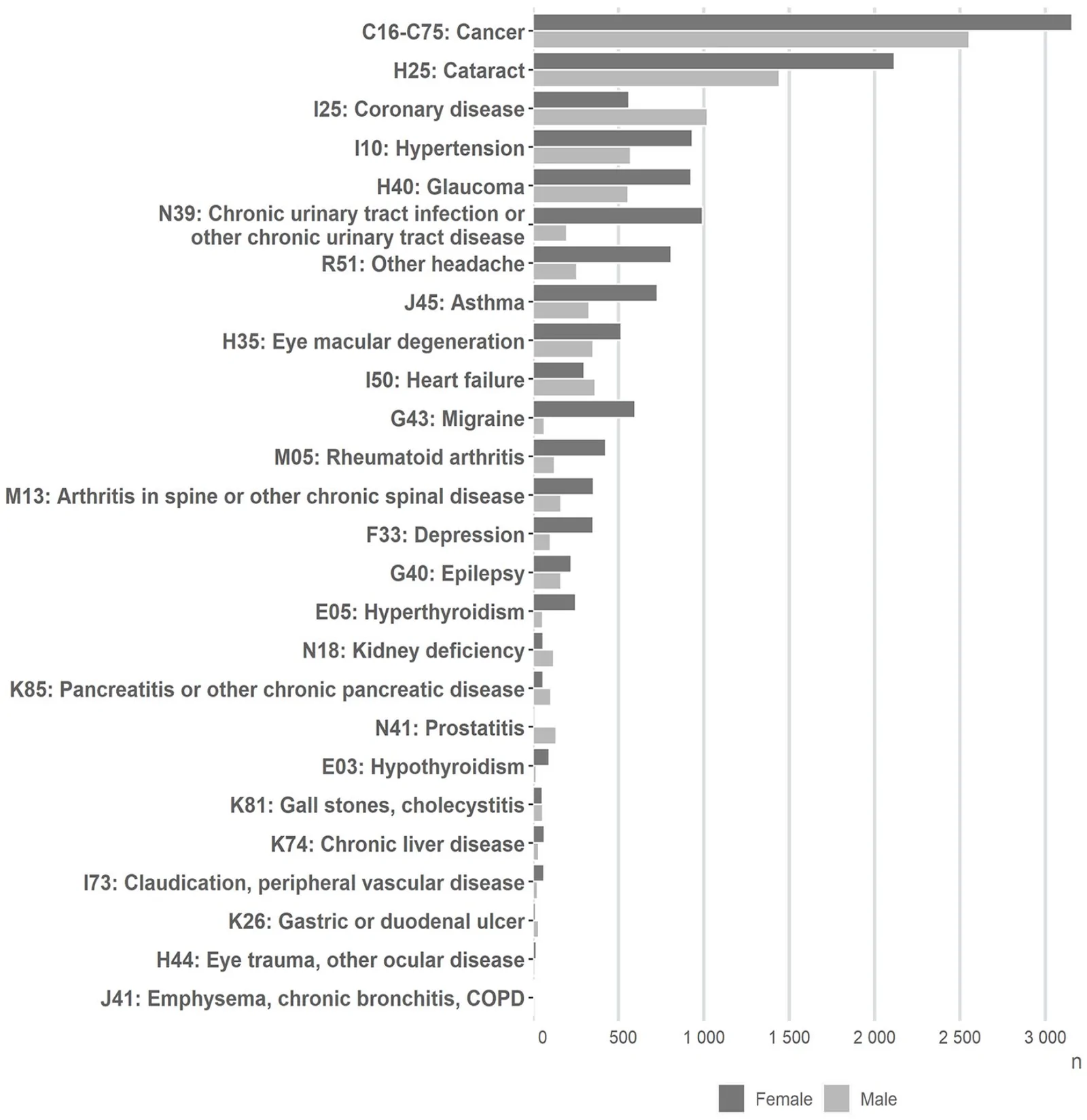
Distribution of diseases in the connecting cohorts with biobanks (CoCoBi) dataset covering sample donors of Biobank Borealis, when the data dictionary disease names from questionnaires were mapped to the respective International Classification of Diseases, 10th revision (ICD-10) codes or code groups.

Biobank Borealis provides a wide variety of hospital data that complement the cohorts’ data collections. Available structured clinical data on consented OUH patients include the following variables: dates of hospital outpatient and inpatient visits from 2008 to the present, type of visit, symptom and cause diagnoses (ICD-10) [22], codes for medical procedures, pati-ent’s municipality, clinic code, age during visit, and date of death if applicable. The pathology tissue samples can be linked with SNOMED codes [24] specifying diagnoses and organ sites. Furthermore, the CoCoBi datasets were enriched by identifying individuals with diagnosis codes related to frailty and ageing [23] (Supplemental Table 2), and by mapping disease names in the data dictionary (based on cohort questionnaires) [21] to ICD-10 codes (Figure 4). Additional structured information, such as laboratory measurements and medication data in Anatomical Therapeutic Chemical(ATC) classification codes [31] can be accessed, as well as unstructured clinical data of OUH patients with biobank consent, which can be manually retrieved from the EHR systems. The data accessible by way of hospital biobanks are predo-minantly retrieved from specialty healthcare units, mostly university hospitals. Currently the availability of primary healthcare data is limited.

Like other Finnish biobanks, Biobank Borealis hosts an accumulating number of genome-wide association study data originating from the FinnGen project [32]. In addition to the diagnostic FFPE tissue archives and the FMC serum collection, which comprise the largest masses of Biobank Borealis’ samples, several other sample types are being prospectively collected (Supplemental Table 1).

## Conclusions

In this project we created a unique resource for ageing-related research, the CoCoBi dataset, using data from three Finnish biobanks. Data from 11 population-based collections were harmonised, containing anthropometric, lifestyle and genomic data, and attributes describing metabolic risk and frailty. The joint pseudonymisation process allowed us to link medical data and clinical samples taken in hospital settings to the original cohort data, enabling new avenues for research into the initial causes of various diseases and disease progression. Following on FAIR principles [27], the CoCoBi datasets composed of existing cohort data were made reusable and interoperable through the harmonisation and joint pseudonymisation process. They are now findable and available for researchers world-wide by way of the Fingenious® Service [28]. Finally, this project provides a model for how to integrate and harmonise different types of datasets across several biobanks, so that they can be jointly accessed and used for research purposes.

## Supplemental Material

sj-docx-1-sjp-10.1177_14034948241228482 – Supplemental material for Connecting cohorts of Finnish biobanks creates a research resource for the study of healthy ageing Supplemental material, sj-docx-1-sjp-10.1177_14034948241228482 for Connecting cohorts of Finnish biobanks creates a research resource for the study of healthy ageing by Niina Eklund, Salla-Maaria Pätsi, Heli Lehtiniemi, Samppa Rohkimainen, Juha Kivelä, Hanna Öhman, Minttu Sauramo, Kyösti Sutinen, Anja Keskinarkaus, Perttu Terho, Tapio Seppänen, Pia Nyberg, Minna Männikkö and Kaisa Silander in Scandinavian Journal of Public Health

sj-docx-2-sjp-10.1177_14034948241228482 – Supplemental material for Connecting cohorts of Finnish biobanks creates a research resource for the study of healthy ageing Supplemental material, sj-docx-2-sjp-10.1177_14034948241228482 for Connecting cohorts of Finnish biobanks creates a research resource for the study of healthy ageing by Niina Eklund, Salla-Maaria Pätsi, Heli Lehtiniemi, Samppa Rohkimainen, Juha Kivelä, Hanna Öhman, Minttu Sauramo, Kyösti Sutinen, Anja Keskinarkaus, Perttu Terho, Tapio Seppänen, Pia Nyberg, Minna Männikkö and Kaisa Silander in Scandinavian Journal of Public Health
