# Supplementary material for: Connecting cohorts of Finnish biobanks creates a research resource for the study of healthy ageing
Source: Scand J Public Health. 2024 Mar 4;54(7):709–17. doi: 10.1177/14034948241228482 (PMC13589791; doi:10.1177/14034948241228482)
Supplement: Supplementary file 1 [file sj-docx-1-sjp-10.1177_14034948241228482.docx]

Supplementary Table 1. Number of individuals in CoCoBi sample collections with different available sample and data categories.

| **Sample collections:**  **Data or sample category:** | **FINRISK (1992-2012)** | **Health 2000 & 2011** | **NFBC 1966** | **NFBC 1986** | **OULU 1935** | **OULU 1945** | **FMC** | **Borealis** |
| --- | --- | --- | --- | --- | --- | --- | --- | --- |
| Participants total | 34570 | 8600 | 11900 | 9390 | 830 | 990 | 988780 | 448980 |
| Sex: men | 16520 | 3790 | 6090 | 4840 | 370 | 550 | 0 | 173810 |
| Sex: women | 18050 | 4810 | 5810 | 4550 | 460 | 440 | 988780 | 275170 |
| Samples: DNA | 34000 | 8600 | 5850 | 6500 | 530 | 990 | 0 | 19180* |
| Samples: serum | 20700 | 8600 | 7790 | 6300 | 530 | 990 | ~2000000^#^ | 21760* |
| Samples: plasma | 20700 | 8600 | 7790 | 6300 | 530 | 990 | 0 | 21760* |
| Samples: cells | 2350 | 0 | 2190 | 0 | 0 | 0 | 0 | 0 |
| Samples: PaxGene tubes (RNA) | 2700 | 0 | 5770 | 2520 | 0 | 0 | 0 | 0 |
| Samples: PaxGene tubes (cfDNA) | 0 | 0 | 0 | 0 | 0 | 0 | 0 | 400* |
| Samples: fecal samples | 0 | 800 | 4250 | 980 | 0 | 680 | 0 | 0 |
| Samples: urine samples | 1320 | 6200 | 4470 | 1010 | 310 | 950 | 0 | 0 |
| Samples: cerebrospinal fluid | 0 | 0 | 0 | 0 | 0 | 0 | 0 | 300* |
| Samples: FFPE tissue samples | 0 | 0 | 0 | 0 | 0 | 0 | 0 | ~1773000 |
| Samples: fresh frozen tumor samples | 0 | 0 | 0 | 0 | 0 | 0 | 0 | 1090* |
| Genomics: array genotypes | 31055 | 7700 | 5400 | 3800 | 0 | 0 | 0 | 0 |
| Genomics: Imputed GWAS | 30620 | 7540 | 5400 | 3740 | 0 | 0 | 0 | 9590 |
| Genomics: WES | 12160 | 4960 | 0 | 0 | 0 | 0 | 0 | 0 |
| Genomics: WGS | 4460 | 200 | 0 | 0 | 0 | 0 | 0 | 0 |
| Omics: NMR metabolomics | 27160 | 8290 | 7690 | 5600 | 0 | 970 | 0 | 0 |
| Omics: RNA expression array | 515 | 0 | 0 | 0 | 0 | 0 | 0 | 0 |
| Omics: methylomics | 510 | 0 | 800 | 550 | 0 | 0 | 0 | 0 |
| Omics: gut microbiome | 6800 | 0 | 880 | 0 | 0 | 0 | 0 | 0 |
| Omics: telomere length | 4070 | 7400 | 7620 | 0 | 0 | 700 | 0 | 0 |
| Biomarkers: lipids | 34570 | 8600 | 7790 | 6580 | 830 | 990 | 0 | In EHR** |
| Biomarkers: environmental toxins | 3100 | 0 | 0 | 0 | 0 | 0 | 3650 | 0 |
| Biomarkers: CRP | 29700 | 8600 | 7790 | 6300 | 310 | 990 | 17300 | In EHR** |
| Biomarkers: liver enzymes | 34570 | 8600 | 7790 | 6300 | 0 | 710 | 0 | In EHR** |
| Biomarkers: glucose | 10800 | 8600 | 7690 | 6180 | 830 | 990 | 0 | In EHR** |
| Biomarkers: insulin | 10600 | 6700 | 7690 | 6580 | 590 | 990 | 0 | In EHR** |
| Biomarkers: vitamin D | 9650 | 8600 | 7670 | 0 | 310 | 650 | 26850 | In EHR** |
| Biomarkers: vitamin B12 | 7650 | 0 | 0 | 0 | 370 | 0 | 3240 | In EHR** |
| Biomarkers: cotinine | 3000 | 7690 | 0 | 0 | 0 | 0 | 16410 | In EHR** |
| Biomarkers: thyroid hormones TSH, T4 and anti TPO | 0 | 6360 | 5790 | 0 | 590 | 990 | 11900 | In EHR** |
| Biomarkers: creatinine | 19490 | 8600 | 5800 | 6300 | 590 | 730 | 0 | In EHR** |
| Biomarkers: infectious diseases (HIV, B-hepatitis, syphilis) | 0 | 0 | 0 | 0 | 0 | 0 | 988780 | In EHR** |
| Information collected during pregnancy | 0 | 0 | 11900 | 9390 | 0 | 0 | 988780 | In EHR** |
| Birth and childhood data | 0 | 0 | 11900 | 9390 | 0 | 0 | 0 | In EHR** |
| Clinical data: blood pressure and pulse | 34570 | 8600 | 7770 | 6780 | 830 | 990 | 0 | In EHR** |
| Clinical data: anthropometrics | 34570 | 8600 | 7790 | 6780 | 830 | 990 | 0 | In EHR** |
| Clinical data: electrocardiography | 0 | 8600 | 5770 | 1780 | 830 | 990 | 0 | In EHR** |
| Clinical data: spirometry | 0 | 8600 | 7700 | 6460 | 0 | 690 | 0 | In EHR** |
| Clinical data: bio-impedance | 10330 | 8600 | 5720 | 1770 | 450 | 990 | 0 | In EHR** |
| Clinical data: OGTT | 10310 | 8600 | 5830 | 0 | 760 | 990 | 0 | In EHR** |
| Clinical data: cognitive tests | 0 | 8600 | 5610 | 1500 | 590 | 980 | 0 | In EHR** |
| Clinical data: dental status | 0 | 8600 | 1960 | 1760 | 590 | 0 | 0 | In EHR** |
| Clinical data: vision tests | 0 | 8600 | 2700 | 1700 | 0 | 0 | 0 | In EHR** |
| Clinical data: auditory tests | 0 | 8600 | 1370 | 1140 | 0 | 0 | 0 | In EHR** |
| Clinical data: strength, balance, reaction time | 0 | 8600 | 5830 | 1800 | 830 | 710 | 0 | In EHR** |
| Background: education*** | 34000 | 8600 | 9410 | 7330 | 830 | 990 | 0 | 0 |
| Background: work & income*** | 34570 | 8600 | 9420 | 2370 | 830 | 990 | 0 | 0 |
| Background: household | 34570 | 8600 | 9410 | 3210 | 0 | 0 | 0 | 0 |
| Background: marital status*** | 34570 | 8600 | 9410 | 3210 | 830 | 990 | 0 | 0 |
| Lifestyle: nutrition | 14700 | 8600 | 9480 | 7730 | 830 | 990 | 0 | In EHR** |
| Lifestyle: smoking | 34570 | 8600 | 9410 | 7330 | 830 | 990 | 0 | In EHR** |
| Lifestyle: alcohol use | 34570 | 8600 | 9410 | 7730 | 830 | 990 | 0 | In EHR** |
| Lifestyle: excercise | 34500 | 8600 | 9430 | 7330 | 830 | 990 | 0 | In EHR** |
| Lifestyle: sleep | 34570 | 8600 | 9410 | 7330 | 830 | 990 | 0 | In EHR** |
| Health status: current health | 34570 | 8600 | 9410 | 7330 | 830 | 990 | 0 | In EHR** |
| Health status: functional ability | 34570 | 8600 | 7650 | 7800 | 830 | 990 | 0 | In EHR** |
| Health status: oral health | 0 | 8600 | 8230 | 7330 | 830 | 0 | 0 | In EHR** |
| Health status: women's questions | 18000 | 4300 | 4840 | 3910 | 590 | 540 | 0 | In EHR** |
| Health status: psychological well-being | 34500 | 8600 | 8310 | 6790 | 830 | 990 | 0 | In EHR** |
| Disease history: cvd*** | 34570 | 8600 | 9410 | 6970 | 590 | 990 | 0 | In EHR** |
| Disease history: lung diseases*** | 34570 | 8600 | 9410 | 6970 | 590 | 990 | 0 | In EHR** |
| Disease history: mental health*** | 34500 | 8600 | 9410 | 6970 | 590 | 990 | 0 | In EHR** |
| Disease history: muscoskeletal symptoms | 34570 | 8600 | 9410 | 6970 | 590 | 990 | 0 | In EHR** |
| Disease history: diabetes*** | 34570 | 8600 | 9410 | 6970 | 830 | 990 | 0 | In EHR** |
| Disease history: cancer*** | 34570 | 8600 | 9410 | 6970 | 590 | 990 | 0 | In EHR** |

*Ongoing collections. Numbers represent the end of 2021.

^#^Sample count from number of unique pregnancies.

**Electronic Health Records (EHR) are available upon request. Some data categories may be incomplete and/or require manual data extraction.

***Additional information for all biobank donors is available from national social and health registers through Findata.
