# Supplementary material for: Connecting cohorts of Finnish biobanks creates a research resource for the study of healthy ageing
Source: Scand J Public Health. 2024 Mar 4;54(7):709–17. doi: 10.1177/14034948241228482 (PMC13589791; doi:10.1177/14034948241228482)
Supplement: Supplementary file 2 [file sj-docx-2-sjp-10.1177_14034948241228482.docx]

Supplementary Table 2. Number of individuals in Borealis Biobank with diagnoses related to frailty and aging

| **ICD code** | **Disease description** | **N** |
| --- | --- | --- |
| **Frailty** |  |  |
| N39 | Other disorders of urinary system | 1189 |
| M25 | Other joint disorders | 1109 |
| R29 | Other symptoms and signs involving the nervous and musculoskeletal systems | 755 |
| R41 | Other symptoms and signs involving cognitive functions and awareness | 713 |
| S01 | Wound of the head | 504 |
| K59 | Other functional intestinal disorders | 466 |
| R33 | Retention of urine | 329 |
| S72 | Fracture of femur | 281 |
| I69 | Sequelae of cerebrovascular disease | 253 |
| E87 | Other disorders of fluid electrolyte and acid-base balance | 243 |
| A41 | Other septicaemia | 226 |
| S00 | Superficial injury of head | 214 |
| I67 | Other cerebrovascular diseases | 180 |
| G30 | Alzheimer’s disease | 152 |
| L03 | Cellulitis | 152 |
| Z73 | Problems related to life-management difficulty | 148 |
| N17 | Acute renal failure | 118 |
| R26 | Abnormalities of gait and mobility | 112 |
| F05 | Delirium | 99 |
| L97 | Ulcer of lower limb, NEC | 83 |
| I95 | Hypotension | 82 |
| R32 | Urinary incontinence | 47 |
| F01 | Vascular dementia | 46 |
| L89 | Decubitus ulcer | 41 |
| E86 | Volume depletion | 38 |
| H54 | Blindness and low vision | 38 |
| G31 | Other degenerative diseases of nervous system | 37 |
| F03 | Unspecified dementia | 23 |
| J69 | Pneumonitis due to solids and liquids | 13 |
| Z75 | Problems related to medical facilities and other health care | 10 |
| R45 | Symptoms involving emotional state | 9 |
| F00 | Dementia in Alzheimer’s disease | 0 |
| B96 | Other bacterial agents as the cause of diseases classified to other chapters | 0 |
| Y95 | Nosocomial condition | 0 |
| W19 | Unspecified fall | 0 |
| W18 | Other fall on same level | 0 |
| **Acute Heart Problems** |  |  |
| R07 | Pain in throat and chest | 2254 |
| R06 | Abnormalities in breathing | 2117 |
| I48 | Atrial fibrillation and flutter | 1761 |
| I25 | Chronic ischaemic heart disease | 1583 |
| I20 | Angina pectoris | 1245 |
| R42 | Dizziness and giddiness | 1240 |
| I49 | Other cardiac arrhythmias | 1229 |
| I35 | Non-rheumatic aortic valve disorder | 775 |
| I21 | Acute myocardial infarction | 704 |
| I50 | Heart failure | 664 |
| R00 | Abnormalities of heart beat | 437 |
| R60 | Oedema | 359 |
| I34 | Non-rheumatic mitral valve disorder | 357 |
| I44 | Atrioventricular and left bundle branch block | 339 |
| Z95 | Presence of cardiac and vascular implants and grafts | 329 |
| E78 | Disorders of lipoprotein metabolism | 290 |
| I71 | Aortic aneurysm and dissection | 261 |
| Z82 | Family history of certain disabilities and chronic diseases leading to disablement | 128 |
| I73 | Other peripheral vascular diseases | 91 |
| J90 | Pleural effusion | 76 |
| I45 | Other conduction disorders | 71 |
| J98 | Other respiratory disorders | 62 |
| I27 | Other pulmonary heart diseases | 37 |
| I51 | Complications and ill-defined descriptions of heart disease | 24 |
| R79 | Other abnormal findings of blood chemistry | 21 |
| I24 | Other acute ischaemic heart diseases | 8 |
| Z92 | Personal history of medical treatment | <5 |
| I08 | Multiple valve disease | 0 |
| **Cancer and Lung Disease** |  |  |
| C78 | Secondary malignant neoplasm of respiratory and digestive organs | 361 |
| C79 | Secondary malignant neoplasm of other site | 330 |
| C34 | Malignant neoplasm of bronchus and lung | 276 |
| J47 | Bronchiectasis | 110 |
| J96 | Respiratory failure | 106 |
| Z51 | Other medical care | 65 |
| J43 | Emphysema | 55 |
| **Elective cataract** |  |  |
| H25 | Senile cataract | 3560 |
| H26 | Other cataract | 736 |
| **Chronic Heart Problems** |  |  |
| I48 | Atrial fibrillation and flutter | 1761 |
| I25 | Chronic ischaemic heart disease | 1583 |
| I20 | Angina pectoris | 1245 |
| Z95 | Presence of cardiac and vascular implants and grafts | 329 |
| Z92 | Personal history of medical treatment | <5 |
